# Advancing Green Gasification—A Review on Biological Pretreatment, Syngas Purification, Machine Learning Technology, and Techno-economic Insights for Biofuel Production

**DOI:** 10.1021/acsomega.5c04385

**Published:** 2025-10-08

**Authors:** Sankar Sudharsan Rameshwar, Santhosh Paramasivam, Natarajan Rajamohan, Brindha Sakthivel, Dhivya Dharshika Kannan, Baskaran Sivaprakash, Gianluca Gatto

**Affiliations:** † Department of Chemical Engineering, 29895 Annamalai University, Annamalai Nagar 608002, India; ‡ Department of Electrical and Electronic Engineering, 3111 University of Cagliari, Cagliari 09123, Italy; § Chemical Engineering Section, Faculty of Engineering, Sohar University, Sohar 311, Oman; ∥ Department of Biotechnology, 231537 Karpagam Academy of Higher Education, Coimbatore 641021, India; ⊥ Tamilnadu Government Polytechnic College, Madurai 625002, India

## Abstract

The dependence on conventional fossils for energy and the ongoing utilization of carbonaceous resources significantly burden the environment. Consequently, researchers have strived to establish sustainable energy-generating methods that employ renewable resources to minimize environmental stress. Gasification of biomass is a potential route to harness the potential of biological reserves. This process strategically employs various agents to catalyze the desired reactions, facilitating the transformation of biomass feedstocks into fuels or alternative products. This article explores various gasification technologies, including catalytic gasification, steam gasification, and supercritical and subcritical water gasification, as a sustainable approach for converting lignocellulosic agricultural residues into biohydrogen. Additionally, comprehensive insights into syngas purification methodologies and carbon sequestration from the produced syngas are presented. One of the key highlights of this review is the utilization of machine learning models for enhancing the efficiency of gasification systems, showcasing the interdisciplinary nature of the research and its potential for future advancements. An economic evaluation is also presented. Implementing supercritical water gasification on lignocellulosic biomass has significantly increased the syngas production rate while decreasing the reaction time. Similarly, in catalytic gasification techniques that employ a variety of metallic and ceramic catalysts, a substantial increase in syngas output has been observed, featuring increased proportions of hydrogen and carbon oxides as well as a complete reduction in tar formation.

## Graphical abstract

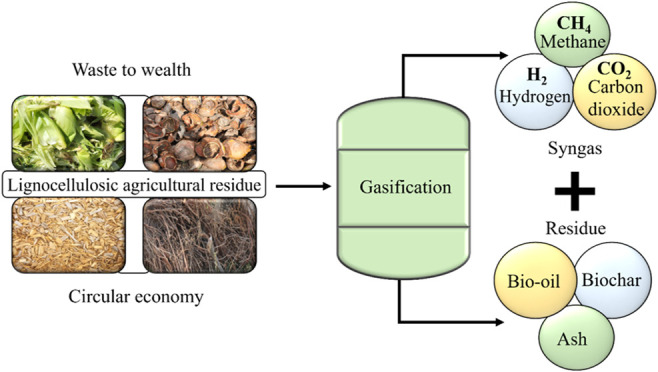

## 1 Introduction

Energy is considered a powering force for developing any rising economies, as it drives industrial advancement and monetary fortunes. By the year 2040, the global energy exigency is expected to spike up in surplus by 28% of current values. Energy harnessed from biohydrogen pertains to the United Nations’ “Sustainability Development Goal,” specifically “Affordable and Clean Energy”.^1,2^ Fuels originating from fossils, including crude oil, coal, and natural gas, have provided power challenges since the beginning of the industrial revolution. However, their exploitation has resulted in detrimental effects on the biosphere from excessive carbon releases. Carbon-neutral approaches, which replace fossils as an energy source, play a chief role in bringing sustainability goals for green and clean energy.^3–5^ Biomass with lignocellulosic richness falls under emerging bioenergy reserves and serves as the capable substitute for crude and petroleum. Lignocellulosic agricultural residues are abundant in many developing countries owing to the high volume of agro-industrial activities in the rural area. However, agricultural residues are hardly valorized and remain untapped. In this context, biomass gasification could be a promising approach to produce biohydrogen and other valorized commodities.^5–7^ Gasification pertains to a thermochemical transition of a carbon-rich substance into gaseous products, including syngas, which comprises hydrogen, oxides of carbon, and methane. The gasification process encompasses a system of interconnected stages taking place inside a gasifier. Subsequently, a series of complex reactions occurs in the reactor named the gasifier under the influence of an oxidizing component at high temperature, whereby the lignocellulosic biomass consisting of lignin, hemicellulose, and cellulose would break down to form syngas. However, biomass gasification also includes the generation of unwanted byproducts comprising tar, sulfur, and nitrogen components.^8,9^ Gasification can be enhanced by optimizing oxidizing and reforming agents. This includes utilizing supercritical and subcritical water and innovative catalytic technologies. Recent studies have prioritized reducing tar formation during the gasification process by building superior catalysts based on metal oxides, metal–organic framework (MOF), core–shell nanoparticles, and ceramic nanomaterials. Similarly, pretreatment of biomass feedstocks can also be conducted to prevent the formation of tar and other undesirable constituents. Comparably, the hydrogen yield can be enhanced by subjecting the yielded syngas to various cleaning strategies such as an electrostatic precipitator and filtration techniques processes.^10,11^ In this review, we have given special interest in making the process greener by fostering biological pretreatment and its impacts on the gasification process. Similarly, this review provides a review of various gasification technologies for bioenergy generation, particularly from lignocellulosic agricultural residues and syngas purification technologies for the enrichment of biohydrogen. This article also focuses on the economic feasibility of biomass gasification initiatives by providing a section for techno-economic analysis of biomass gasification and gives an insight into futuristic approaches involving machine learning (ML) for the optimization of gasification processes.

## 2 Biological Pretreatment of Lignocellulosic Agricultural Residue for Enhanced Gasification

Biological pretreatment is regarded as a sustainable approach and has gained worldwide attention as it is not expensive, not energy intensive, and does not utilize any harmful chemicals. Unlike physical, chemical, and thermochemical pretreatment, the biological pretreatment does not cause any secondary pollution.^12,13^ Biological pretreatment uses the ligninolytic property of various microbes, which include bacteria, yeast, and fungi, to deconstruct cellulose, hemicellulose, and lignin that exist within the biomass. This improves cellulose accessibility during the gasification process and significantly reduces the tar formation and energy intensity of the complete procedure.^14–16^

In research, biogas slurry with various microbial consortium and nutrients was utilized as a microbial source to pretreat corn straw before gasification. This method improved the overall process and increased the lower heating value by 1.15 MJ/(N·m^3^) and reduced the presence of tar by 21% with minimal dry matter loss. The utilization of industrial and domestic waste enriched with nutrients not only pertains to the circular economy but also improves the total thermal efficiency of the process.^17^ Similarly, in research led by Chen et al. 2019, the team subjected the biomass to a 14-day anaerobic digestion (AD) prior to the 800 °C air gasification process. This facilitated a partial breakdown of complex organic matter and improved the feedstock reactivity and overall conversion. As an effect, the gasification produced high-quality syngas, achieving a carbon conversion of 73.62% and lowering heating value by 6.83 MJ/(N·m^3^). The anaerobic digestion also reduced the level of tar formation by 35% and eliminated any operational challenges pertaining to the formation of tar. Moreover, the techno-economic feasibility studies proved that this integrated approach can reduce the overall process by 44% and increase the net profits by 25% compared to conventional biomass gasification.^18^

In conclusion, the integration of bio-pretreatment together with gasification enhances the overall process efficacy, reduces tar formation, and enriches the biohydrogen content in syngas as depicted in Figure 1. Basically, bio-pretreatment improves sustainability by utilizing the microbes grown from the industrial and domestic waste, pertaining to the waste-to-wealth concept and promotes the circular economy.^19–21^ Extracting fermentable sugars and other extractives from the bio-pretreatment procedure adds values to the overall process and can trade off its drawbacks, which include slow reaction rates and sugar losses.^5,22,23^ Moreover, these drawbacks can be focused on and optimized through future studies and research, supporting sustainable and feasible bioenergy production.

**Figure 1:**
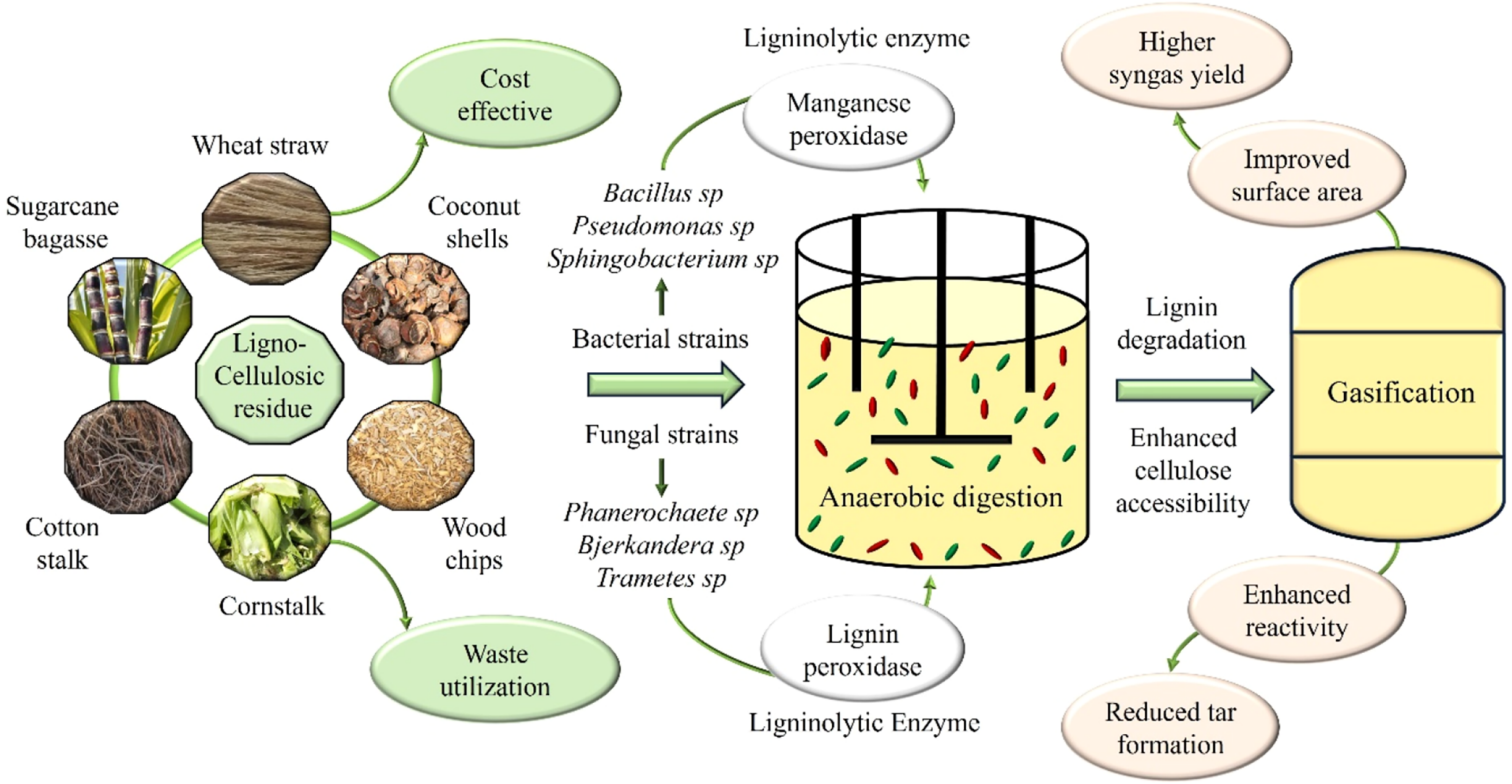
Anaerobic digestion of lignocellulosic biomass using bacterial and fungal strains to degrade lignin enhances cellulose accessibility for gasification.

## 3 Gasification Technologies to Produce Biohydrogen from Lignocellulosic Agricultural Residue

Gasification involves the conversion of lignocellulosic and other biological components through a thermochemical route to generated hydrogen-enriched syngas and other valorized products. Gasification involves the fractional oxidation of biological components at high temperature conditions in attendance of a catalyst or agents which includes steam, catalyst, air, or oxygen. This partial oxidation of biological substances yields syngas comprising methane, hydrogen, and oxides of carbon and other hydrocarbons. Among the various gasification routes, steam gasification can produce hydrogen-rich gas with high cold gas efficacy (the ratio of energy content, typically the lower heating values of the produced syngas to that of the input feedstock). Similarly, catalytic gasification can improve the hydrogen yield and reduce tar formations. Supercritical and subcritical gasification provide a viable alternative by utilizing water’s unique properties at different temperature and pressure ranges to deconstruct biomass components into valorized commodities as provided in Figure 2.

**Figure 2:**
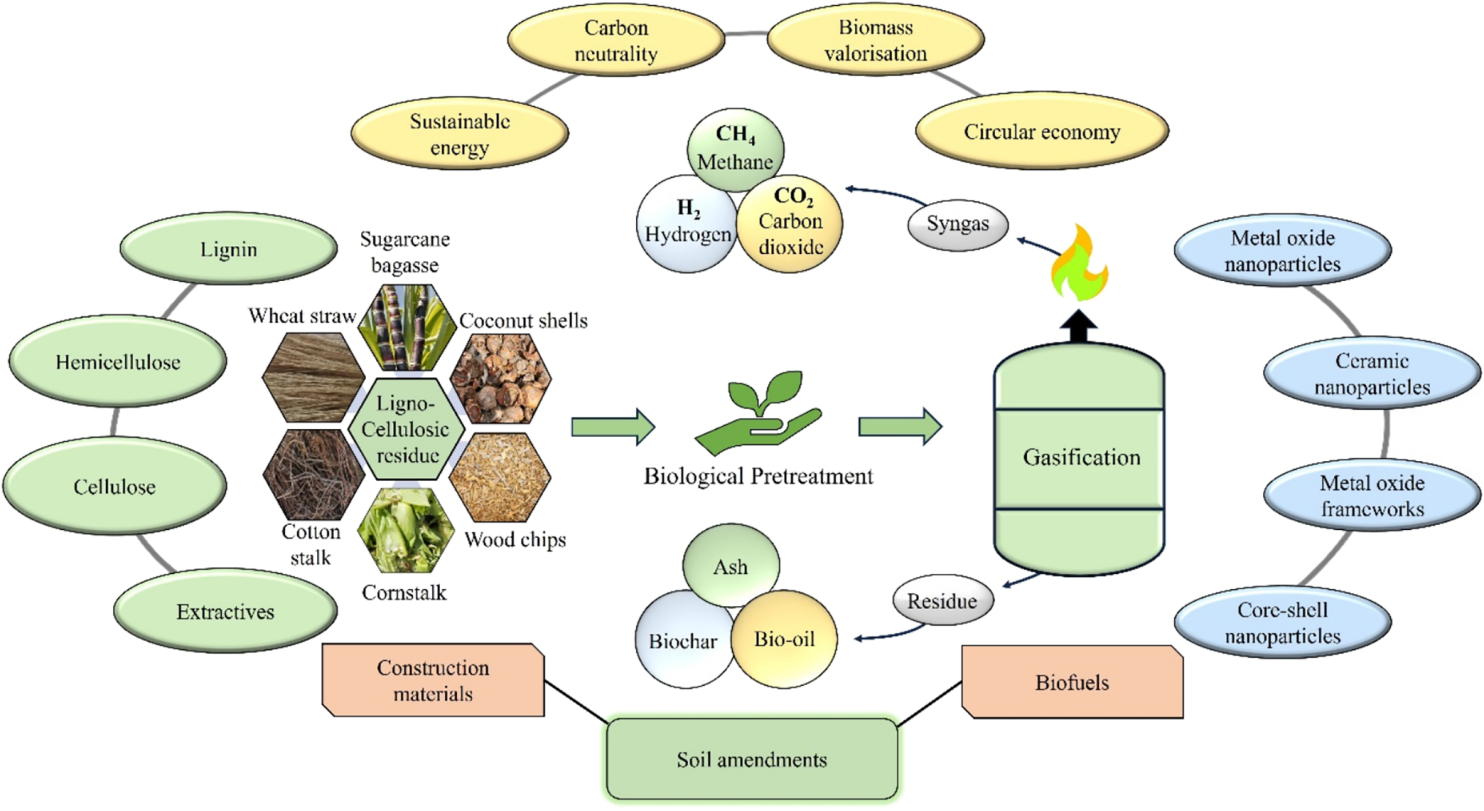
Valorization of lignocellulosic biomass through biological pretreatment and gasification.

### 3.1 Steam Gasification of Lignocellulosic Agricultural Residue

A robust methodology was established to produce biohydrogen from different kinds of biomass. The Gibbs energy equation was applied to rectify the errors from the prior models, and the thermodynamic laws governing steam gasification were analyzed. After these corrections, an experiment produced H_2_ from rice husk. This experiment decreased the general errors of thermodynamic nonstoichiometric and stoichiometric simulations from 4.53 to 2.46 and 2.89 to 2.36, consequently. The final parameters influencing steam gasification by wood include moisture content, the temperature of gasification, and the ratio between steam and biomass.^24^ The hydrogen generation from rice husk employs an Air Separation Unit (ASU) with oxygen content ranging between 85 and 99%, serving as a gasifier. The efficiency ratio of gasification ranged from 2 to 4, restoring the cold gas efficacy. Cold gas efficiency (CGE) of 70% and 107.8 kg of hydrogen per dry biomass were obtained using 95% oxygen with a gasification ratio of approximately 4.0. The gasifying equivalence ratio (ER) is directly proportional to the energetic efficiency. However, the ASU experiences energy destruction and losses in the overall chemical reaction.^25^

Furniture waste was employed as a lignocellulosic biomass for biohydrogen generation using a catalytic gasification process. A novel solvent-deficient method utilizing 10 wt % nickel-loaded catalysts was employed, with catalysts including Al_2_O_3_, CeO_2_, CeO_2_–La_2_O_3_, and CeO_2_–ZrO_2_. The hydrogen selectivity (vol %) of the catalysts followed the descending order: Ni/CeO_2_–ZrO_2_ exhibited the highest selectivity, tailed by Ni integrated CeO_2_, then Ni integrated Al_2_O_3_, and finally Ni integrated CeO_2_–La_2_O_3_, which had the lowest selectivity. Due to smaller proportions of nickel crystals, nickel was finely distributed on the catalyst exterior, and the Ce_1–*x*_Zr_*x*_O_2−δ_ solution, the Ni integrated CeO_2_–ZrO_2_ catalyst, exhibited superior catalytic activity. The aforementioned bifunctional chemical reaction has significant commercial value for biomass gasification.^26^

Hydrogen production by steam gasification was examined by using Ni-loaded biochars such as unrefined char, steam-pretreated char, and nickel/α-Al_2_O_3_. The steam-treated nickel char showed superior gasification efficacy as compared to others. The mesoporous structure of the resulted coke was confirmed through the characterization analysis. The silica composition within the chars offers mechanical support for nickel stacking and prevents the formation of coke.^27^ A small-scale steam gasification unit was organized utilizing palm kernel shell (PKS). The upgraded PKS process was used to determine optimized conditions to synthesize H_2_ production and syngas gasification by ensuring a coal-based catalyst obtained from bottom ash. To obtain the final results, the process must be done with the conditions of catalyst size of 1–2 mm, a gasification temperature of 625 °C, and a percentage of 7.5% between coal, coal ash, and palm kernel shell. A model of the developed gasification plant was simulated using Aspen Plus software, and the results were confirmed using the obtained empirical data.^28^

The research based on hydrogen generation by air-steam gasification was exhibited by the NiO/γ-Al_2_O_3_ nanocatalyst. The various parameters of this experiment were measured by the biomass ratio. In the process of making hydrogen, the NiO/γ-Al_2_O_3_ nanocatalyst was used in the cracking of tar and hydrocarbons and in refining the gas quality. The higher temperature applied is directly proportional to the high yield of hydrogen production. The varying equivalence ratio (ER) in the gasification process exhibits a result of 0.22, as noted in the current study. The steam against biomass ratio provided the optimal value of 1.33 in the gasification operation.^29^ The gasification process utilized cashew shells and husks and a fluidized bed (FB) reactor at a temperature of 750 °C. The presence of a catalyst was determined by characterization studies, and the highest hydrogen was 8.1%, with a lower heating value of 4.3 MJ/m^3^, while at an equivalence ratio of 0.35, the cold gas efficiency and carbon transition energy were observed to be 54.9 and 91%, respectively, for the rice husk combined with corn stalk.^30^ Biohydrogen is produced from food waste using a catalyst containing nickel and aluminum combined with eggshell, and homo. Steam gasification generated more hydrogen than air gasification, which created more CO_2_-rich gases. At 800 °C, H_2_-rich gases with low C_2_–C_4_ concentration were formed, resulting in gaseous products with 59.48 vol % hydrogen. The equal spreading of nickel species on the exterior of aluminum oxide pellets increased their catalytic activity, stability, and selectivity.^31^

Corncob (40 kW, without grains) was widely utilized in biomass gasification, yielding 1500–5500 million tons annually. With high volatile characteristics, the downdraft gasification process produced a tar content of 80% DAF. The syngas composition was assessed to study the yield of hydrogen, the cold gas efficacy, and the lower heating value. The hydrogen output was 310 N·mL/g, the lower heating value was 4.8 MJ/(N·m^3^), and cold gas efficacy was 59.18% using steam and calcium carbonate. Mathematical research revealed that calcium carbonate and steam boosted hydrogen generation by 15.8 and 10.8%, respectively. The criteria were reached using a calcium carbonate/biomass ratio of 1% and a ratio between steam and biomass of 11% (w/w). CGE increased by 5% with steam, 10% with CaCO_3_, and 10% due to the synergy between steam and CaCO_3_.^32^ CeO_2_-modified bifunctional nickel catalyst, modified using the sol–gel technique, served as the primary catalyst in this gasification process. At 500 °C, the catalyst increased H_2_ concentration to 85.81 vol %, yielded 35.82 mmol/g of biomass, had a steam-to-carbon biomass molar ratio of 5 and catalyst to biomass mass ratio of 2.5, and had the lowest level oxides of carbon formations. The prepared catalyst possessed outstanding cyclic stability in H_2_ production, CO_2_ sorption, and carbon deposition inhibition. During 10-cycle testing, the concentration and yield of hydrogen remained above 81.88 vol % and 32.11 mmol/g biomass, respectively.^33^

### 3.2 Catalytic Gasification of Lignocellulosic Agriculture Residue

The research aims to operate a pilot horizontal gasifier of rubberwood pellets and eucalyptus wood chips to produce syngas with an H_2_/CO ratio of 1.8–2.3 for Fischer–Tropsch (FT) synthesis. It is divided into two parts: temperature and gas product. The alternative method uses the pilot horizontal gasifier because of the optimum temperature and tar removal from the CaO ratio between steam and biomass on the resulting H_2_ to CO ratios. The laboratory-scale gasifier applied raw biomass feeds to determine the optimum gasifying temperature. The tar level was below 0.2 wt % with calcium oxide as the reforming catalyst. At 900 °C, a pilot horizontal gasifier with a steam-to-biomass ratio of 0.5 and 0.2 wt % CaO loading was operated to produce H_2_ and CO in a ratio of 2.0. For rubberwood pellets and eucalyptus wood chips, this gave rise to constant syngas composition, output rates of 2.5 and 2.7 kg/h, and H_2_/CO proportions of 1.8 and 1.9.^34^

A highly effective method for turning wet biowaste, including food scraps, paper waste, sewage sludge, agricultural trash, and forestry residue, into hydrogen-rich syngas was supercritical water gasification (SCWG). In the biowaste conversion sector, the catalytic SCWG is a potential technique for producing hydrogen. SCWG biowaste is assessed by using metal-based catalysts. Rare earth oxide-promoted catalysts, noble metal bimetallic catalysts, noble metal-free bimetallic catalysts, and alkaline earth metal-promoted catalysts were added to the list of metal-based catalysts. In the SCWG’s presence, metal-based catalysts were created to solve technical issues and potential future applications. It leads to outstanding catalytic performance on physiochemical characteristics and their uses in the SCWG of biowaste.^35^ In a pilot-scale updraft gasifier, researchers used several gasification agents in their experiment, including air, a steam with air combination, and a catalyst. The best ways to increase H_2_ generation from rice husk gasification were found to be the H_2_ concentration, minimal heating value, and H_2_/CO ratio in syngas. Adding dolomite in air gasification results in a high H_2_ level in the resulting gas, as high as 15.4 mol %, followed by 7.08 and 3.6 mol % if steam/air and air alone are used as gasifying agents. Consequently, an airflow velocity of 3 m^3^/h and a catalyst blending ratio of 15% were found to be the optimal working conditions.^36^

The effect of CaO on volatile gasification was investigated under various conditions. The experiment was conducted in a two-stage fixed-bed pyrolysis–gasification setup. As a catalyst and CO_2_ absorber, CaO accelerated volatile breaking and gasification reactions and encouraged the shift in chemical equilibrium, which produced more H_2_. It is advised to use a temperature between 600 and 700 °C and a water injection rate of 0.1 g/min. The H_2_ emission in this study is closely related to the biomass materials’ volatile content.^37^ When rice husk is gasified catalytically in a fluidized bed, H_2_-rich gas is created. With the highest H_2_ concentration of 69.16 vol % and yielded 11.86 mmol/g rice husk, the process is optimized for effective carbon conversion and generation of hydrogen at a calcium-to-carbon molar ratio of 1.5, a steam-to-carbon proportion of 1.5, a gasification rate of 500 °C, and a catalytic transformation temperature of 800 °C. When fresh Ca­(OH)_2_ is supplied during calcination, hydration activates the regenerated CaO. The Ce–Ni/γ-Al_2_O_3_ catalyst improves the hydrocarbon reforming processes of WGS, SMR, and C_2_–C_3_. When it comes to maintaining H_2_ concentration and yield, the Ce–Ni/γ-AlO_3_ catalyst demonstrates remarkable stability.^34^

A research study explored the potential of a cost-effective catalyst made from alkaline earth minerals through a systematic method conducted through a response surface methodology. Here, the relationship between the loaded catalyst and the iron content on gasified yield was studied, and it was observed that the catalyst had a high affinity for iron oxide, calcium ferrite, and calcium oxide. Across all tested conditions, the results demonstrated improved char conversion, gas generation, and gasification efficacy. However, an increase in the level of tar formation was also observed. Under optimized conditions, the study achieved a gasification efficiency of 69.57%, a total gas yield of 402.8 mL/g of biomass, and a char yield of 24.68 wt %. These results suggest the potential of a prepared low-cost catalyst in converting waste biomass to various value-added commodities.^38^ Similarly, researchers from Argentina studied the valorization of waste derived from Patagonian rosehip using a CO_2_-supported gasification system. The rosehip husk waste exhibited a strong affinity for the gasification process, requiring less activation energy and higher reactivity. Furthermore, the incorporation of rosehip husk ash into orthoasphaltite char led to a reduction in the gasification temperature by up to 200 °C, thereby confirming its catalytic role. These results highlight the promising potential of rosehip waste as a viable feedstock for biomass gasification, not only underscoring its value but also emphasizing the effectiveness of biomass-derived ash as a sustainable catalyst in char-based gasification systems.^39^ Table 1 presents the results of biohydrogen production from various lignocellulosic agricultural residues, using a catalyst.

**Table 1:** Enhancement of Biohydrogen Production from Various Lignocellulosic Agricultural Residues Using a Catalyst^a^

feed	catalyst/reactant	temperature (°C)	H_2_ yield	methods used	references
sugar beet pulp and HPDE as plastic waste	Ni–Cu/AC-Cao	385–460	49.62 and 33.74 mmol/g	co-gasification	^40^
wheat straw (WS)	Fe_2_O_3_/Al_2_O_3_	950	0.67–1.53 L/g	chemical looping gasification	^41^
acid hydrolyzed waste wheat	hydraulic retention time (HRT)	90	VHPR = 1.75 L H_2_/L and *Y*_H_2__ = 1.6 mol	hydrolysis	^42^
corncob char	salts of KOH, K_2_CO_3_, NaOH and Na_2_CO_3_	900	197.8 g/kg	catalytic steam gasification	^43^
*Cymbopogon citratus*	Ni/dolomite/CeO_2_/K_2_CO_3_	600–1000	81.01 g/kg	steam air gasification	^44^
eucalyptus	nickel-loaded Thai brown coal char	500–650	46.68 mol	catalytic steam gasification	^45^
rubberwood pellets and eucalyptus wood chips	Cao	900	2.5 and 2.7 kg/h	steam–biomass gasification	^46^
food residues, paper scraps, sewage, agriculture, and forestry residue	zinc, nickel, cobalt, and chromium	540	15 mol/kg	supercritical gasification	^35^
pilot-scale rice husk (3 kg)	dolomite		9.6 mol %	updraft gasification	^36^
calcium oxide Cao/O	pyrolytic volatiles	600–700	61.83 vol % and 210.97 mL/g	absorption-enhanced steam gasification	^37^
rice husk	Ce–Ni/γ-Al_2_O_3_ and Ca(OH)_2_	500	11.67 mmol/g	staged catalytic gasification	^34^

a NA—not available.

### 3.3 Supercritical Water Gasification of Lignocellulosic Agriculture Residue

The continuously flowing apparatus for gasification by supercritical water (SCWG) of alkaline is being researched. It is being evaluated at temperatures between 400 and 600 °C and 25 MPa, having residence times between 4.94 and 13.71 s. When residence time grew, the temperature rose, but when gasification occurred, it decreased. With the maximum chemical oxygen demand (COD) elimination effectiveness of 88.69% observed at 600 °C, the hydrogen content ranged from 40.26 to 61.02%. The COD process has a pseudo-first-order reaction with an activation energy of 74.38 kJ/mol and a pre-exponential factor of 104.05 s^–1^.^47^ Agricultural wastes, including lignocellulosic biomass, are important bioenergy resources. Agricultural wastes such as straw from wheat, walnut outer shells, and almond shells were studied in SCWG in Iran’s Kurdistan Province, adopting a 26 mL stainless-steel batch microreactor. The total gas output increased until it steadied after 30 min. After 10 and 20 min of reaction time, the hydrogen yields from grain straw, almond outer shells, and walnut shells were 7.25, 4.1, and 4.63 mmol/g, respectively.^48^ SCWG is a novel black liquor (BL) treatment that attempts to minimize pollutants and recover energy. Co-gasification with wheat straw increased energy supply in the pulping process while optimizing alkali utilization. The optimal BL/WS mixing ratio of 1:1 yielded the highest gasification efficiency through a synergistic effect. Increasing temperature improved gasification efficiency, achieving a maximum carbon gas efficiency of 97.87% at 750 °C for the BL/WS mixture.^49^

The SCWG evaluated rapeseed hull, food, and straw, common agricultural residues in Canada, as alternative fossil fuel sources. These residues were hydrothermally processed at 350–500 °C for 20–80 min at 23–25 MPa of pressure, yielding elevated hydrogen levels ranging from 7.1–8.1 mmol/g. With a high-hydrogen output of 29.7 mmol/g after optimization at 500 °C, 60 min, and 10 wt %, SCWG is a growing hydrothermal technique for lignocellulosic biomass reduction.^50^ CaO adsorption and a water-based gas shift converter enhanced SCWG’s hydrogen production. 99.99% hydrogen was generated by the SCWG of bagasse in conjunction with CaO adsorption. Using steam and supercritical water agents, biomass (bagasse) gasification outperformed SCWG-CaO in terms of energy utilization and hydrogen production. Because of its low greenhouse gas emissions and environmental friendliness, biohydrogen might replace conventional energy sources.^51^ SCWG was utilized to produce high-hydrogen syngas from grain straw, walnuts, and almond outer shells in a do-it-yourself batch microreactor system. The rates of hydrogen gas production from grain straw, shells of walnuts, and almond shells were 6.52, 4.26, and 4.1 mmol/g, correspondingly. Because of its considerable cellulose and hemicellulose content, wheat straw produced the most hydrogen and carbon gasification, with efficiencies of 42.6 and 46.9%, respectively.^48^

SCWG was used to test the synthesis of syngas from corncob and sisal waste. Compared to sisal waste, which had higher ash and moisture, corncob had higher volatile matter and less moisture. Compared with jute waste, the corncob exhibited a higher thermal value. Light hydrocarbons, CO, CH_4_, and H_2_ are all present in the resultant syngas. Syngas was deemed suitable for oxo-synthesis if the H_2_/CO proportion was less than 1. The potential of biomass conversion as a sustainable energy source was shown by statistical analysis with an *R*-square value of 0.992 for corncob and 0.944 for sisal waste.^52^ A number of crude treatments were contrasted with SCWG. Three possibilities were considered: 10% Ni–0.08% Ru/Al_2_O_3_–ZrO_2_ catalyst, uncatalyzed feedstock, and 10% Ni–0.08% Ru/Al_2_O_3_. After 5 h, the highest hydrogen outputs were 193 and 187%. The disintegration of oxygenated hydrocarbons, with a mean absolute variance of 3.55%, was the rate-limiting phase, according to a mechanistic model based on Eley–Rideal.^53^

Solid waste is converted into hydrogen-rich gaseous products using SCWG technology. The rate-limiting process for the gasification of wheat straw in SCW is identified by using a kinetic model. The generation of hydrogen increased with temperature, reaching 7.2 mol/kg around 100 °C.^54^ Although there has been little investigation, SCWG-based hydrogen-rich syngas synthesis is renewable. Using analytical methods, maize stover was examined under SCWG conditions. Aliphatic compounds were initially gasified and hydrolyzed at 400–500 °C. Essentially, less than 10.5/100 g of water was determined to be the optimal SCWG concentration.^55^

### 3.4 Subcritical Gasification of Lignocellulosic Agriculture Residue

This study explores the hydrothermal gasification of enriched hemicellulose from white poplar sawdust, examining the impact of temperature (300–600 °C) and catalysts on product yield. Hydrothermal gasification presents a sustainable approach to managing agricultural and industrial waste. The results reveal that isolated hemicellulose yields more gaseous products and less solid residue than sawdust. Additionally, the gasification of isolated hemicellulose outperformed xylose and natural biomass with regard to product yield. These findings underscore the potential of hemicellulose-rich biomass for efficient gasification and valuable product generation.^56^

As the energy demand, emissions of greenhouse gases, prices for gasoline, and availability of fossil fuels decline, biofuels made from lignocellulosic materials are becoming increasingly popular. In order to produce hydrogen, this study gasified farm waste, specifically soybean and flax straw, in subcritical (300 °C) and supercritical (400–500 °C) water. Particle dimensions (0.13 and 0.8 mm), residence time (30–60 min), biomass-to-water proportion (1:5 and 1:10), and temperature (300–500 °C) were all examined at 22–25 MPa. A high of 6.62 mmol/g of H_2_ was generated by soybean straw at 500 °C, 1:10 in addition, 0.13 mm size, and 45 min of exposure. Hydrogen, carbon dioxide, and methane outputs were improved by the KOH catalyst. Supercritical water gasification shows promise for efficient H_2_ production from waste biomass.^57^

An ecologically friendly and energy-efficient method of lowering biomass recalcitrance is microwave pretreatment. Miscanthus (*Miscanthus giganteus*) and switchgrass (*Panicum virgatum* L.) were large-yielding perennial grasses that may be used to produce biofuels and other bioproducts. This study discovered that after microwave pretreatment at various temperatures, the biomass of switchgrass and miscanthus was dissolved in subcritical water. Aqueous-phase reformation (APR) was used to evaluate the hydrolysates for the production of hydrogen-rich gas. By decreasing biomass recalcitrance, higher microwave temperatures raised its solubility in subcritical waters by 7–10%. In contrast to untreated biomass, pretreated biomass hydrolysates produced fewer gaseous products when gasified. Miscanthus produced the greatest amount of gas from unprocessed material because it was more responsive to microwave treatment. The quantity of ungasified carbon solid leftover in the APR operation was enhanced by pretreatment.^58^ With a focus on the impact of the initial quantity of water on the composition of gas and hydrogen production at varying temperatures, this study examined the gasification of the Kenaf feedstock under subcritical liquid–vapor conditions. Using varying water quantities, temperatures, and reagents (Ru/C, Pt/C, Na_2_CO_3_, Fe_2_O_3_, CaO, CaCO_3_, RuCl_3_), gasification was conducted in a batch reactor. The findings showed that the initial amount of water significantly affected both hydrogen selectivity and gasification efficiency. Using water as both a reactant and a reaction media, the RuCl_3_ catalyst produced the most carbon conversion (71.3%) and the best H_2_ selectivity (44.5%) at 250 °C.^59^ As a way to fulfill rising energy demands and cut greenhouse gas emissions, biofuels made from lignocellulosic material are becoming increasingly popular. This work produced hydrogen fuel from wheat straw via hydrothermal gasification. Sub and supercritical water phases were examined. Investigations were conducted to investigate the consequences of temperature, input concentration, and response time. To maximize the yields of hydrogen and total gas, two metal catalysts—Ru/AlO_3_ and Ni/Si–AlO_3_—were investigated. At 550 °C, 20 wt % feed amount, and 60 min, supercritical water gasification increased hydrogen yields (2.98 mmol/g) and overall gas yields (10.6 mmol/g). Hydrogen yields rose to 5.1 mmol/g and total gas emissions to 18.2 mmol/g using catalytic gasification.^60^ Table 2 presents the details on biohydrogen production using lignocellulosic biomass through gasification.

**Table 2:** Biohydrogen Production Using Lignocellulosic Biomass through Gasification Procedure along with the Operating Conditions

feedstock	reactor used	H_2_ yield	catalyst used	preparation	temperature (°C)	reference
wheat straw	fluid temperature reactor	11.26 kg/mol	alkali in black liquor	soda pulping	600	^47^
wheat straw, walnut shell, almond shell	stainless-steel microreactor	6.52, 4.26, 4.1 mmol/g	NA	P. J. Van Soest	440	^48^
wheat straw	nickel-based alloy	46.02 kg/mol	alkali salt	soda pulping	750	^49^
wheat straw, canola meal, timothy grass	316 stainless-steel reactor	2.15 mmol/g	K_2_CO_3_ and 20Ni–0.36/Al_2_O_3_	coprecipitation	650	^61^
canola hull, meal, and straw	tubular batch reactor	8.1, 29.7 mmol/g	NA	NA	500	^50^
cellulose, xylan, lignin	water	NA	K_2_CO_3_	biochemical and thermochemical methods	550	^62^
hydrocarbon	water gas shift	3.52 mmol/g	NA	biomass gasification	750	^51^
cattle manure, corn husk	SCWG reactor	1.65 mol/g	10% Ni–0.008% Ru/Al_2_O_3_–ZrO_2_	Eley–Rideal method	900	^53^
wheat straw	quartz tube batch reactor	12.88 mol/kg	NA	lumped parameter	700	^54^
corn stover	SCW reactor	NA	NA	rapid pyrolysis method	600	^55^
sawdust	NA	NA	NA	alkali	600	^56^
kenaf	micro hydrothermal gasification reactor	NA	RuCl_3_	steam reforming method	250–300	^59^
switchgrass, miscanthus	stainless-steel micro bench reactor	NA	NA	APR, micro pretreatment method	120	^58^
pinewood, wheat straw	fixed-bed reactor	2.8–5.8 mmol/g	nickel	hydrothermal water gasification	300–500	^63^
wheat straw	tubular reactor	2.98, 10.6 mmol/g	hydrogen	NA	550	^60^
soybean and flax straw	fixed-bed-based tubular reactor	6.62, 14.91 mmol/g	KOH	Gibbs free minimization method	500	^64^
wheat straw	fluid temperature reactor	11.26 kg/mol	alkali in black liquor	soda pulping	600	^47^
wheat straw	nickel-based alloy reactor	46.02 kg/mol	alkali salt	soda pulping	750	^49^

## 4 Recent Advances in Syngas Purification Technologies for Biohydrogen Enrichment

Syngas purification is an evolving field that deals with separation of the desired output from the mixture of components present within the syngas. Recent advances of syngas purification techniques include cryogenic distillation, which separates gases based on their freezing point, and adsorption process employing emerging materials like graphene aerogel, metal–organic framework, and other metallic nanoparticles, which enhance the hydrogen purity. In this section, we will be focusing on recent methodologies studied for the separation and purification of syngas to enrich the biohydrogen yield. A cryogenic-based module was developed by using heat exchangers to produce a hydrogen-enriched stream. The cryogenic process utilized a refrigeration cycle as well as an additional nitrogen refrigeration stream. Heat exchangers accounted for more than 88.4% of exergy destruction. The refrigeration capability of nitrogen increased by 10.60% throughout this procedure.^65^

A biological process called “dark fermentation” turns organic waste into biohydrogen. Biohydrogen separated from fermentation generally comprises 30–40% of CO_2_. It is the most effective procedure for hydrogen enrichment, with a 90% recovery rate. It also lowers particular expenses in high-vacuum processes. A two-stage carbon technique may readily be used to purify biohydrogen. A lower specific cost of United States Dollar (USD) 0.06/N·m^3^ was predicted to achieve a 99.5 vol % biohydrogen purity, making it more economical than pressure swing adsorption (PSA).^66^ This work used carbon molecular sieve (CMS) membranes made from cellulose hollow fiber precursors to separate H_2_/CO_2_. Asymmetric carbon membranes performed well with a humidified mixed gas supply. Carbon membranes were less effective in producing over 99.95% H_2_ with negligible hydrogen loss until operated at higher temperatures. A two-stage H_2_-selective carbon membrane device may readily achieve H_2_ purity of more than 99.5%.^67^ Zeolite 13X can separate hydrogen from a multicomponent bioderived gas. It adsorbs significantly in syngas, with nonisothermal breakthrough and thermodynamic characteristics. The H_2_ production reactor transforms carbon monoxide to CO_2_, which is subsequently separated from hydrogen using adsorption. CO_2_ does, however, result in a modest desorption. A thorough parametric study was carried out concerning feed flow rate and pressure. A new technology for hydrogen purification was developed, and the adsorption stability performance was validated in a prototype plant for 1500 cycles.^68^ A viable substitute for producing hydrogen is biomass gasification. Hydrogen production from syngas may be increased by improving the H_2_/CO ratio for renewable methanol synthesis. The carbon hollow fiber membranes’ capacity to separate in binary as well as quaternary H_2_-containing mixtures of different compositions was carefully examined. Carbon monoxide and nitrogen are frequently found in syngas. The syngas membrane was optimized to concentrate hydrogen and convert biomass into biofuel. A feasibility analysis of carbon membrane separation found that optimizing the H_2_/CO ratio may remove 80–90% of N_2_.^69^ This procedure purifies hydrogen and separates CO_2_ from the hydrogen-based synthesis produced by single-cycle vacuum pressure swing adsorption (VPSA). The VPSA original is increased during the CO_2_ separation to regulate the Cu-TDPAT in the adsorption process. It requires a continuous separation procedure for zeolite 13X Cu-TDPAT, which is a potential adsorbent for H_2_ purification with CO_2_ capture. This study has shown how a thorough, fundamental understanding of the relationship between process and material qualities may result from combining process and material layout investigations.^70^ An actual syngas flow from the ethanol transformation was evaluated for CO reduction. AuCu/CeO_2_ catalysts’ catalytic activity is increased by the CeO_2_ nanostructure. CeO_2_ with lattice plane shapes of {111} and {100} shows strong activity. {110} planes increase the CO_2_ selectivity, although they become unstable when used for extended periods. An AuCu/CeO_2_ catalyst with a polyhedral structure was used to produce hydrogen suitable for fuel cells. These findings lead to advances in hydrogen generation.^71^ To produce hydrogen, biomass sources must be optimized for effective energy storage. A two-stage hydrogen purification method extracts a high-purity hydrogen from syngas. The highest H_2_ concentration was found using the Taguchi approach. To boost the production of hydrogen, the ratio of steam-to-carbon monoxide (S/CO) was adjusted. For H_2_ concentration in adsorption-based vacuum-driven swing operations, the reaction between the water and gas shift works well. At 2 kg/cm^2^ pressure and a flow rate of 17 L/min, the optimized process produced 93.61% H_2_ purity, 31.63% H_2_ recovery, 4.86 mol H_2_/(kg_ads·h) productivity, and an energy expenditure of 448.14 kJ/kg H_2_.^72^ Membrane plus cryogenic distillation methods were used to study hydrogen separation. Tests were conducted by using membrane-supported (Case I) and cryogenic-supported (Case II) techniques. In both situations, hydrogen was initially generated from syngas. Waste heat from the water–gas shift reactors was collected and incorporated into an organic Rankine cycle. With Aspen Hysys v11, membrane plus cryogenic distillation procedures were modeled. Due to the high cost of the necessary compressors, economic research showed that the first scenario was more costly (USD 17.7 million) than the second scenario (USD 10.2 million). For applications using hydrogen energy, hydrogen purification is essential. Two popular techniques for removing hydrogen from various gas mixtures are vacuum-powered swing adsorption (VPSA) and pressure swing adsorption (PSA). PSA using an AC5-KS adsorbent performed better than VPSA. The impact of feed duration on the recovery of hydrogen was examined, and then it was shown that longer feed times reduced hydrogen purity while greater recovery rates increased purity. For improved performance, the artificial neural network (ANN) approach is used for hydrogen purification. The best result was obtained when hydrogen recovery reached 88.65% after 223 s of feeding and 96 s of purging.^73^ As hydrogen gains importance as a clean energy carrier, more research and technical breakthroughs in syngas purification will be required to fulfill the rising demand for high-purity hydrogen in industrial and energy applications.

In summary, methods for purifying hydrogen can be broadly categorized as physical, chemical, or mechanical processes. Each technique is selected based on the type of contaminants present and the production scale. The two most common techniques are pressure swing adsorption (PSA) and membrane-based separation; the former is less preferred for purification due to its high energy requirements and associated costs. In turn, considering the tendency toward lower operating costs and a smaller environmental impact, membrane technology is thought to be more sustainable. Despite producing high-quality, ultrapure hydrogen, cryogenic separation is not a viable alternative for routine operation, given its high-power consumption and major energy loss. Advanced adsorption materials like metal–organic frameworks (MOFs), carbon molecular sieves, and zeolite 13X, as stated in this section, emerge to be suitable choices for the selective removal of CO_2_ and other trace gases, offering frequently high yield and cost-effectiveness. For industrial requirements, those methods do necessitate multistage procedures in most high-temperature scenarios. Although membrane-based processes present a unique variety of economic and environmental benefits, in order to reduce methane slip, future research must concentrate on resolving challenges with membrane durability, compatibility with different gas compositions, and contamination from different raw biogas stream contaminants.^74,75^

## 5 Machine Learning Approach for Improved Gasification Process

In this work, four machine learning models were used to forecast and evaluate a simulation involving the supercritical water gasification process. These models assessed the efficacy of hydrogen production in supercritical water gasification and described the inner workings of the optimal model. With a coefficient ratio of 0.9782 in hydrogen yield, the random forest (RF) model performed better than the support vector machine, neural network modeling, and Gaussian process regression models. According to the RF model contour plots, the maximum hydrogen conversion rate of 45.6% and exergy efficacy of 43.3% were obtained by using feedstock with high levels of oxygen and a low hydrogen-to-carbon ratio. The model makes it clear that, in experimental or real-world engineering settings, parameters associated with the supercritical water gasification procedure for hydrogen production depend on suitable feedstock and parameter optimization.^76^

Six units make up the biomass gasification-based hybrid energy cycle (HEC): two for the production of electric energy, one for heat recovery, one for the electrolysis of water to create hydrogen, one for the generation of thermal power, and one for the manufacture of biofuel. Energy, exergy, and exergoeconomic concerns are all incorporated into the conceptual analysis. The combustion chamber receives excess hydrogen to enhance efficiency and lessen dependence on conventional energy sources. Biofuel production is predicted using machine learning techniques such as support vector machines and Gaussian regression methods. The HEC generates around 71.8 kmol/h of hydrogen, 10.2 MW of electricity, and 153 kW of thermal power. Gaussian process regression is not as effective as that of support vector machines. Remarkably, increasing the gasification pressure does not affect the production of biofuel.^77^

The surplus energy generated by the integrated power cycle powers heating, cooling, and ammonia storage by using hydrogen and biofuels. Ammonia is produced, and hydrogen is stored by using a new integrative biomass gasification energy system. Warm air that comes from solar panels is fed through a gasification machine to provide fuel for the electricity generation. After being heated to the electrolysis temperature, cooling water breaks down. The hydrogen that is created is kept in reserve for use in the production of ammonia fuel. A gas turbine unit and a Rankine cycle make up the power cycle, and exhaust gas is sent to a double-effect absorption chiller’s cooling cycle. It is possible to generate as much as 7 MW of power with a 66% energy efficiency. The rates of hydrogen storage and ammonia synthesis are 0.101 and 0.38 kg/s, respectively. With about 64% energy efficiency, machine learning and genetic algorithm optimization produce 0.5347 kg of ammonia every second.^78^

A two-stage gasification process turns low-value resources, such as garbage, into hydrogen or syngas, while providing precise control. Operating conditions and materials have a considerable effect on gas output and characteristics. A neural-network-based model combined with experimental data enhances the procedure while saving money and time. The gas composition is accurately predicted by the model (*R* ^2^ > 0.99). Highest carbon conversion, hydrogen production, and low CO_2_ emissions are the goals of optimization. The first and second stages’ predicted temperatures (900 and 1000 °C, respectively) and the steam/carbon ratios (3.8 for nitrogen and 5.7 for carbon dioxide) match the experimental findings. Gas output, hydrogen, and carbon dioxide concentrations were 96.2 wt %, 70 mol %, and 16.4 mol % for nitrogen and 97.2 wt %, 66 mol %, and 12 mol % for carbon dioxides at the highest possible levels.^79^ Estimates of hydrogen production through biomass co-gasification plus polymers are made using machine learning algorithms. The production of hydrogen is the dependent variable, whereas the sizes of the plastic and biomass particles, feed proportions, and temperatures are the independent factors. The relative significance ratings of the independent variables are as follows: temperature > proportion of plastics > RSS particle dimensions > high-density polyethylene (HDPE) particle size. Surface-controlled response is correlated with size dependence. The production of hydrogen is more affected by size than by temperatures around 500 and 900 °C. Train-test split, cross-validating, and GridSearch CV models were used to make predictions on unknown data; gradient boosting performed better than the others.^80^

Supercritical water gasification transforms biomass into charcoal and hydrogen-rich gas. The high computing costs of steady-state analysis with the computing fluid dynamics impede dynamic investigations. Based on partitioning theory, a reactor network model reduces dynamic simulation time from days to seconds, including flow, heat transport, and kinetic dynamics; open-loop simulations using the RNM produce data sets for recurrent neural network (RNN) development. While closed-loop optimization is sparse, open-loop simulations are now the focus. A framework for ML-based asymmetric predictive control and an artificial learning model is created. The RNN’s model predictive control maximizes the generation of carbon and hydrogen. The open-loop data show a decrease in H_2_ production from 0.00022 to 0.0002 kg/s when the temperature drops from 700 to 600 °C. To reach the set point, MPC raises the temperature to 708 °C and the mass flow rate to 417.57 kg/h. By adjusting the temperature to 475 °C and the mass flow rate to 407.38 kg/h, MPC may enhance carbon yield and decrease CO_2_ yield.^81^

This study uses artificial intelligence and Gibbs minimization to investigate catalytic impacts on gasified yield. A delta term measures the catalytic impact on the Gibbs free energy. In addition to the Gibbs minimization approach, an artificial neural network improves gasified yield estimation. The ANN model links the catalyst properties to the outlet value. This integration solves a problem in standard thermodynamic models by neglecting the catalyst impact. The ANN model’s A mean squared deviation of 0.042 and *R* ^2^ score of 0.92 indicate excellent prediction accuracy. The hybrid GM model increases the prediction of hydrogen yield by 98% mean squared error (MSE) and 93% overall mean error when compared with the basic model. According to sensitivity research, hydrogen production rises with greater temperatures and lower pressure, particularly at lower biomass contents.^82^ Figure 3 shows the applications of deep learning and machine learning models in biomass gasification for process optimization. It illustrates the role of neural networks and decision algorithms in improving efficiency, dynamic control, and automation. The input layer includes process parameters, while hidden layers refine the predictions. The output layer yields optimized parameters, cost analysis, and life cycle improvements, enhancing energy-saving strategies.

**Figure 3:**
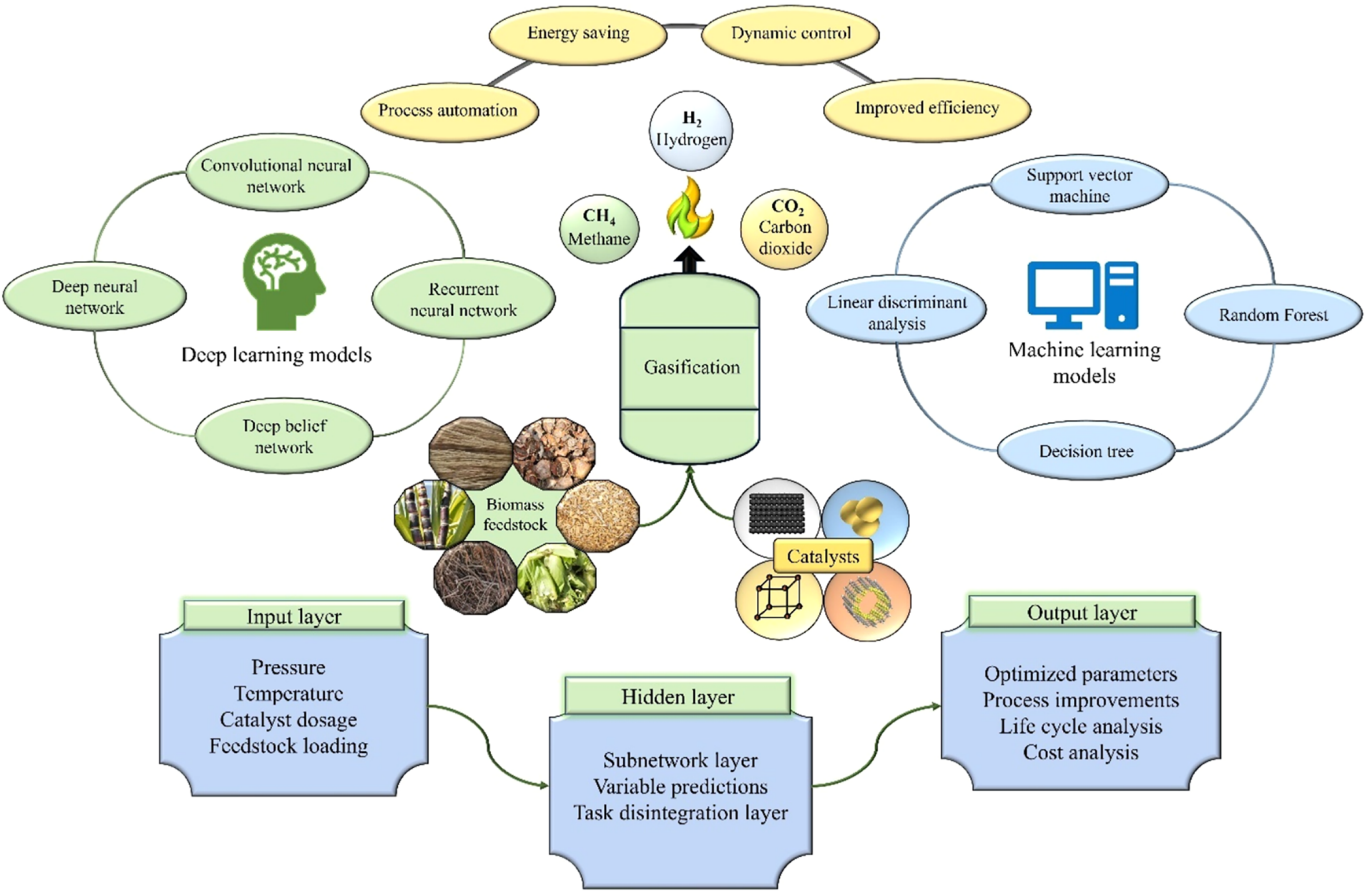
Deep learning and machine learning models are integrated into biomass gasification for process optimization.

In summary, analysis, forecasting, and optimization of biomass gasification processes have benefited greatly from machine learning. In that regard, various models, such as Random Forest, Support Vector Machines, Neural Networks, Gaussian Process Regression, and Gradient Boosting, were used for gas yield prediction, operational parameter optimization, and efficiency enhancement. According to reviewed studies, the Random Forest model achieved the most accurate hydrogen yield prediction (*R* ^2^ = 0.9782) based on optimal feedstock conditions and process settings. However, the model’s performance differed according to the application setting. For example, when combined with Gibbs minimization, ANN demonstrated significant predictive skills, especially when it came to estimating catalytic impacts. RNNs were employed for dynamic optimization using model predictive control, although primarily in open-loop simulations. Temperature, particle size, and feed ratio were identified as important criteria determining hydrogen generation by using sensitivity studies. Across applications, ML models reduced simulation time, increased process efficiency, and enabled cost-effective optimization; nonetheless, computing demands and the need for large, high-quality data sets remain constraints. The use of ML in gasification improves system automation; yet, real-time, closed-loop solutions are still in their early phases, necessitating more development.^83,84^

## 6 Techno-economic Assessment for Gasification

Biowaste and other carbonaceous materials may be converted into valuable products through the beneficial process of gasification. Recent research has focused on the procedure’s sustainability and economic viability, with the goal of making gasification technology commercially viable and lucrative. This can be achieved by integrating the gasification process into various industrial operations, such as integrated heat and power systems or solar-driven hybrid gasification systems. This section pertains to the techno-economic assessment of biomass gasification to study the feasibility of the method.

The system was utilized to produce electricity at low grades. Indium oxide–indium slurry (oxygen carrier) was used in chemical looping gasification. In the gasification process, the operating mode was controlled by thermodynamic models. In the resultant gas (coproduction of syngas), the molar ratio was determined by the liquid metal against feedstock and steam to feedstock proportions. Due to the partially self-sustained property of this system, it does not produce CO_2_. To maximize energy, the synthetic gas is partitioned at LMO/C = 0.1 and S/C = 1.5, resulting in a syngas quality of around 1.55. Peak performance may be maintained under almost isothermal circumstances. The energy lost between reactors is minimized, while power blocks are maximized. The techno-economic investigation found a cheap price. The normalized cost of electricity was constrained to match existing energy sources based on several cost scenarios.^85^ A discounted cash-flow study was conducted to evaluate small-scale H_2_ generation. Solar energy was the major source of hydrogen generation and was used in thermal and hybrid processes as an alternative to traditional techniques. A 50% decrease in heliostat and solar tower expenses enabled the three processes to converge. The weakened autothermal process limited biomass feedstock, whereas hybrid solar and solar-only gasification performed better. The research found that solar gasification is more successful than nonsolar techniques for lowering CO_2_ emissions. Future cost reductions in solar gasification are expected as solar energy becomes more widely adopted, eventually decreasing the process’s standardized cost.^86^

The migration of biomass gasification in solid particles was studied to estimate the standard energy output. Techno-economic feasibility was used to evaluate integrated heat and power in gasification of biomass. Integrating heat and power plants reduced fuel usage while producing 100 tons/day of biomass gasification. The analysis revealed that the resultant energy cost was USD 0.0035/kWh less than that of biomass-only operations. Hybrid gasification outperformed prior approaches, with return rates that were 2.41 and 1.3 times higher, respectively. The coupling of heat and power plants was critical for biomass gasification, and the carbon credit price had a substantial influence on economic viability. The sensitivity of the heat selling price affects economic consequences.^87^ The steam-integrated gasification of coconut coir pith and char was modeled by ASPEN-based simulations. A sensitivity study was performed at 1023, 1073, 1123, 1173, and 1223 K, using steam-to-feed ratios of 0.5, 1.0, 1.5, 2.0, and 2.5. The process was optimized at 1123 K with a steam-to-feed ratio of 0.75, which produced positive results. Steam gasification effectively generates syngas, boosting H_2_ and CO while lowering CO_2_ and CH_4_. In comparison to crude coir pith gasification, AlNouss et al. found that coir pith char gasification produced a 25% greater H_2_ + CO composition and 27% more syngas.^88^

Biomass gasification is a clean and long-lasting way to generate hydrogen. The well-defined Aspen Plus model divided biomass gasification into three major categories: (1) conventional gasification, with steam-to-biomass ratios less than 3; (2) supercritical gasification, with green hydrogen concentrations ranging from 15 to 25%; and (3) plasma gasification. Supercritical gasification has emerged as the most promising option for producing green hydrogen. Green hydrogen had minimum selling prices of Euro (EUR) 7/kg, EUR 10/kg, and EUR 13/kg. Despite the many gasification-based hydrogen generation techniques, further study is necessary to produce green hydrogen at a reasonable cost.^89^ A technique for carbon capture during hydrogen generation is provided by Bioenergy with Carbon Capture and Storage (BECCS). Economic feasibility was determined using techno-economic analysis (TEA) and life cycle assessment (LCA) of producing hydrogen in the western United States, mostly by gasification. When compared to hydrogen obtained from fossil fuels, hydrogen derived from forest leftovers is more affordable (USD 1.52–2.92/kg H_2_). For a 2000 dry short ton/day plant, self-sufficient energy designs reduced the environmental effect and produced a carbon-capturing cost of USD 75/tonne of CO_2_. Consequently, BECCS helps to reduce the environmental effect and decarbonize the process.^90^

In the UK’s West Midlands, technologically viable biomass production may supply 22,000 tonnes of hydrogen annually for more than 2000 public transport vehicles. Steam reforming, rapid pyrolysis–steam reforming, fluidized bed (FB) gasification, and rapid pyrolysis–FB gasification of biogas following anaerobic digestion (AD) all produced hydrogen. The payback period for the first investment varied from 5.10 to 7.18 years. The lowest hydrogen pricing was USD 3.40/kg from the FB gasification method, followed by USD 4.20/kg from AD-biogas reforming, USD 4.83/kg from pyrolysis-reforming, and USD 7.30/kg from pyrolysis alone. Hydrogen produced by FB and AD-biogas reforming was less expensive (USD 4/kg) than CO_2_-based hydrogen (USD 7/kg) under carbon capture. Thus, it was decided that it was possible to produce biomass for public transportation in Birmingham plus the neighboring West Midlands area.^91^ A techno-economic examination of soybean straw identified many pretreatment options for producing hydrogen by water gasification. The study involved around 56,000 metric tonnes of soybean straw every year. The break-even cost of hydrogen was calculated to be USD 1.94/kg (excluding storage and transportation), which is the minimal selling price. The net rate of return was 37.1%, implying poor hydrogen production rates. Nonetheless, this technology was regarded lucrative and economically viable due to its cheap cost.^92^ Similarly, in the sub-Saharan region, there exists a promise for the integration of biomass and solar energy to attain a sustainable energy supply.^93^ Researchers have successfully engineered a reliable method to produce electricity by combining a photovoltaic cell and a small diesel generator with a collective heat and power generation unit, thereby converting readily available agricultural waste into syngas. The study reveals that the system achieved an overall efficiency of 62 and 93.8% in CO_2_ reduction. From economic considerations, even by conservative estimates, the system showed great promise for long-term viability, with a projected total profit of about USD 157,890 over the 20-year operational life of the system and hence a payback period of slightly below 7 years. The levelized cost of electricity (LCOE) was estimated at USD 0.287 per kWh, which is very much in agreement with values reported in similar research.^94^

Beyond the great Savannahs of Africa, South America, especially the Amazon region, holds great potential for biomass gasification due to the large availability of staple crops that produce considerable agricultural residue when grown.^95^ Researchers undertook a field assessment of the gasification of cassava residues from northeastern Brazil through a mathematical model, making it possible to simulate and optimize the process under realistic operating conditions and equipment constraints. Model predictions were validated through laboratory-scale experiments. Syngas production of 2.22 N·m^3^/kg of residue with a lower heating value of 3.917 MJ/(N·m^3^) and 80% cold gas efficacy was obtained at an equivalence ratio of 0.35. The study further forecasts that Northeastern Brazil may yield about 5.81 billion N·m^3^ of syngas from the 2.61 million tons of cassava residues generated every year.^96^

Moving to Southeast Asian countries, where agriculture is considered as the backbone of the economy, generates a massive amount of agricultural waste that may be repurposed as a sustainable source of energy.^97,98^ In Bangladesh, roughly 4 million tonnes of rice straw are openly burnt, posing a huge environmental danger.^99^ To tackle this, researchers from Bangladesh presented a techno-economic feasibility study of producing energy from filtered rice straw. Based on simulations and experiments, it can be estimated that rice straw of 8640 tonnes can generate 217.21 GWh of power per year, but Bangladesh generates 119.3 million tonnes per year, which shows a tremendous prospect. A twin-fire fixed-bed gasifier was simulated using ASPEN PLUS, where a reduction of CO_2_ emission in amount of 0.03% was attained. The study recommends setting up small- to medium-scale rice straw-based power plants to boost rural energy access and promote sustainable development, with an annual cost of USD 4,082,005 in the engineering design and an opportunity cost of USD 0.019 per unit.^100,101^

Green hydrogen can be produced, and CO_2_ can be efficiently removed by biomass gasification. To get 99.95% hydrogen concentration and 90% carbon capture efficiency, a number of pretreatment methods were examined, such as membrane filtering, physical and chemical scrubbing (absorption), and hybrid membrane-chemical scrubbing. This technique produced great efficiency, ranging from 57 to 59%. According to Calin-Cristian Cormos (2023), membrane-based CO_2_ collection has expenses of 7% CAPEX, 7–9% OPEX, and 7% hydrogen. Long-haul biomass gasification generates large amounts of pure hydrogen. The viability of clean hydrogen generation was assessed using biomass type, availability, and proximity to hydrogen markets in the United States. Prices for producing hydrogen using a 40 t/day plant using a novel gasification process were calculated to be USD 3.47/kg H_2_ in Klamath County, OR, USD 4.11/kg H_2_ in Park County, CO, along with USD 3.63/kg H_2_ in Middlesex County, MA.^102^ Cook et al. found that the length of the pipeline had a substantial impact on biomass gasification economics.^103^

According to reviewed techno-economic studies, the cost-effectiveness of biomass gasification is mostly determined by process integration, resource availability, and smart feedstock utilization. Processes that maximize energy recovery, such as integrating heat and power cycles, using solar-assisted systems, and implementing isothermal operations, greatly minimize energy losses and operating costs. For example, chemical looping gasification using indium oxide slurry reduces CO_2_ emissions and maintains consistent performance, providing environmental and economic benefits. Hybrid systems that combine solar energy with gasification show additional cost savings, particularly when construction expenses for heliostats and towers are reduced. Smart biomass utilization, such as employing agricultural wastes like soybean straw, cassava waste, and rice straw, converts low-value or waste materials into high-value energy products. This reduces feedstock prices while simultaneously addressing waste management and environmental problems. Region-specific evaluations, including those conducted in South America, Southeast Asia, and Africa, reveal that utilizing locally available biomass can drastically reduce hydrogen production costs while improving energy access. The integration of carbon capture technology, as well as the smart colocation of resources, increases economic returns. Overall, cost-effective biomass gasification relies on optimizing system design, judiciously utilizing abundant waste feedstocks, and aligning technology with regional resource assets, as shown in Figure 4, to generate clean, affordable energy.^104,105^

**Figure 4:**
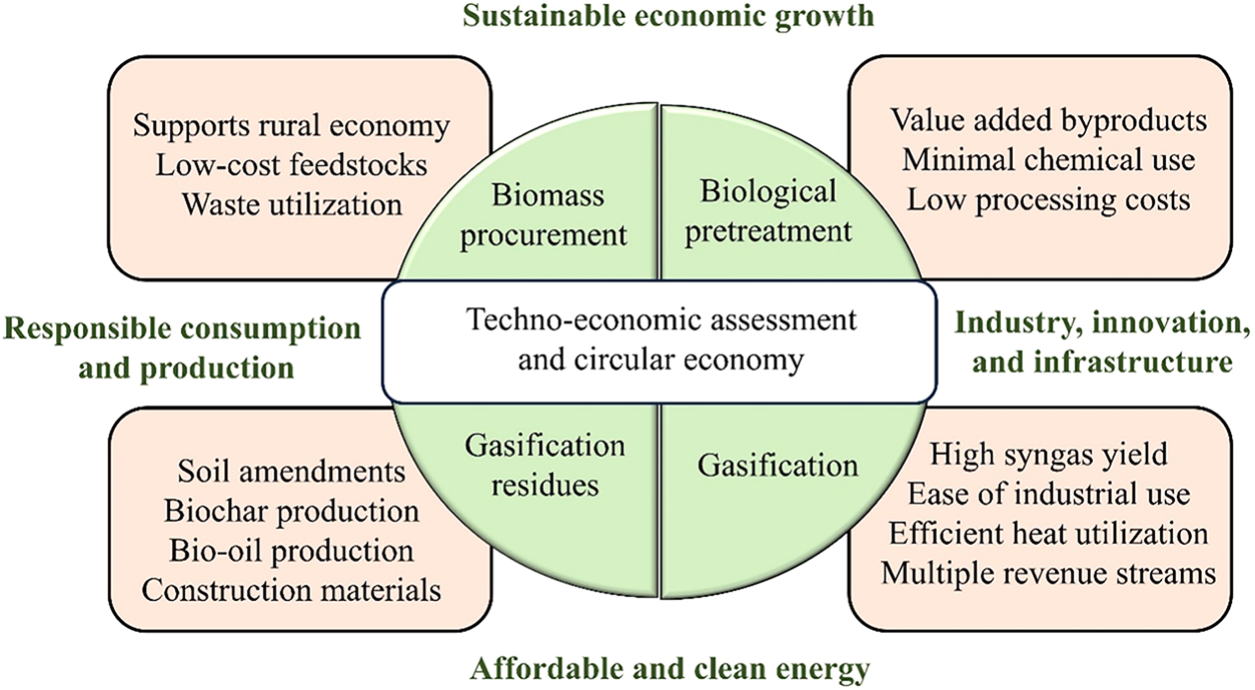
Techno-economic evaluation framework for enhanced biofuel production.

## 7 Challenges, Future Directions, and Conclusions

The change toward a circular economy will require continual improvements of biomass valorization and bioenergy storage strategies and integrated novel technologies to maximize the efficacies. Future studies should prioritize the improvement in feedstock pretreatment, optimizing gasifier design, and enhancing product recovery.^106,107^ Adopting automation and machine learning technologies can facilitate more efficient and scalable bioenergy generation. The policy framework should be strengthened to promote the valorization of agricultural residues and industrial byproducts, thereby encouraging the rural economy to adopt the waste-to-wealth concept and accelerating the shift from fossil to renewable biobased alternatives. Integration of biowaste to the gasification process aligns with the carbon-neutral approach and toward the sustainable developmental goals.^108,109^

The gasification process integrated with biological pretreatment offers a holistic route to utilize various biological substances in the form of both feedstock and microbial sources. This also enhances the overall process by improving the biohydrogen yield and reducing tar formations. Future research should emphasize cultivating microbial strains to enhance the deconstruction of robust characteristics of lignocellulosic biomass and developing a scalable integrated bioprocess that allows for consistent product yield with effective energy recovery.^110,111^ Similarly, industrial waste from the steel and mining sectors can be effectively applied for the manufacture of catalysts and oxygen carriers that can promote the gasification process. However, their reliability and performance efficacy should be studied.^112,113^

A multidisciplinary approach is needed to optimize the gasification process, particularly in understanding reaction kinetics and the performance of the catalysts. Machine learning techniques such as neural networks and decision tree algorithms can change how data are analyzed.^84,114^ By utilizing artificial intelligence, researchers can predict reaction behavior in a more accurate way and can make biohydrogen production a practical approach. Similarly, process intensification can be adapted to have a higher hydrogen yield and a reduced carbon footprint. Strategies such as tar cracking, CO_2_ capture, and syngas purification can improve energy recovery, and the application of novel reactor configurations, including compact catalytic reactors and supercritical water gasification, can be promising for bioenergy generation.^115,116^

Most biomass gasification systems stay at laboratory or pilot size owing to the difficulties inherent with scaling up the technology. High capital investment, challenging operational conditions, and a lack of supportive legislative frameworks are significant impediments to industrial-scale implementation. Furthermore, reliable biomass collection, preprocessing, and transportation pose significant logistical challenges, especially in rural and underdeveloped areas. To address these concerns, future research should prioritize the development of standardized procedures for biomass procurement and pretreatment. Ensuring homogeneous feedstock composition and removing contaminants will improve the system efficiency and repeatability. Regional design innovations that utilize locally available agricultural residues, such as rice straw in Bangladesh or cassava waste in Brazil, offer economically feasible and self-sustaining solutions.^95,96,101^ These approaches reduce transportation costs and can be tailored to meet the needs of rural communities. Similarly, merging biomass gasification with existing renewable sources, such as solar energy, as demonstrated in savannah regions (discussed in the previous section), paves the way for sustainable and decentralized energy systems.^94^ The use of machine learning increases the possibility for process optimization by allowing the discovery of high-performance combinations.^83^ Policy incentives, modular plant designs, and region-specific resource utilization will be critical in overcoming scalability barriers. Together, these measures can help biomass gasification become an economically viable and environmentally friendly energy alternative.

In conclusion, the integration of the circular economy into bioenergy is essential for achieving sustainable resource utilization. Gasification, anaerobic digestion, and chemical looping processes provide a viable pathway for converting biomass and industrial waste into valuable energy products. Advancements in biotechnology, catalysis, and machine learning can further enhance these processes, making them more efficient and cost-effective. Moving forward, interdisciplinary collaborations and policy support are crucial in scaling up bioenergy production while minimizing environmental impacts. By improving conversion techniques, increasing process efficiencies, and lowering operating costs, bioenergy may play a crucial part in the worldwide shift to a carbon-neutral economy.
